# Feasibility of Different Exercise Modalities for Community-Dwelling Residents With Physical Inactivity: A Randomized Controlled Trial

**DOI:** 10.1097/jnr.0000000000000578

**Published:** 2023-10-25

**Authors:** Yu-Hsuan CHANG, Shiow-Ching SHUN, Min-Hsin CHEN, Yin-Fan CHANG

**Affiliations:** 1 PhD, RN, Assistant Professor, Department of Nursing, National Tainan Junior College of Nursing, Tainan, Taiwan; 2 PhD, RN, Professor, Institute of Clinical Nursing, College of Nursing, National Yang Ming Chiao Tung University, Taipei, Taiwan; 3 MS, Assistant Professor, General Education Center, National Tainan Junior College of Nursing, Tainan, Taiwan; and Doctoral Student, National Taiwan Sport University, Taoyuan, Taiwan; 4 MD, Assistant Professor, Department of Family Medicine, National Cheng Kung University Hospital, College of Medicine, National Cheng Kung University, Tainan, Taiwan.

**Keywords:** body composition, endurance training, high-intensity interval training, lipids, resistance training

## Abstract

**Background:**

Exercise interventions can promote health, but they can be difficult to implement. Moreover, no consensus has been reached regarding which exercise modality promotes the most significant improvement in metabolic health.

**Purpose:**

This feasibility study was conducted to (a) determine the implementation efficacy of supervised and home-based exercise interventions by investigating their respective rates of intervention adherence, adherence to targeted intensity, attrition, and adverse events and (b) explore the preliminary efficacy of 12-week exercise programs among aerobic exercise, aerobic exercise combined with resistance exercise, and high-intensity interval training on body composition, anthropometric parameters, and lipid profiles for community-dwelling residents with physical inactivity.

**Methods:**

This randomized controlled trial was conducted from April to October 2020. Seventy-two sedentary participants aged 40–70 years were enrolled and randomized into one of four groups: 12-week aerobic exercise, aerobic exercise combined with resistance exercise, high-intensity interval training, and control. The three exercise groups performed at least moderate-intensity supervised exercise twice a week and home-based exercise once a week, whereas the control group maintained their usual daily activities. The target variables, including body composition, anthropometric parameters, and lipid profiles, were measured before and after the 12-week intervention.

**Results:**

The intervention adherence rates were 74.01%–87.54% for the supervised exercise group, 64.98%–83.90% for the home-based exercise group, and 82.65%–92.65% for the target exercise intensity group. The attrition rate ranged from 12.50% to 17.65%, and no adverse events were reported in any of the exercise groups. Preliminary efficacy data show the reductions in body weight (95% CI [0.01, 1.20], *p* = .048) and low-density lipoprotein (95% CI [2.76, 30.32], *p* = .02) were greater in the exercise groups than the control group, although the intergroup differences were not significant.

**Conclusions/Implications for Practice:**

Body weight and low-density lipoprotein may be efficiently reduced in a 12-week period using any of the three exercise modalities with at least 82.65% adherence to moderate-intensity exercise and 70.84% adherence to exercising 3 times a week.

## Introduction

Exercise promotes metabolic health and helps prevent obesity-related diseases (Ryan et al., 2020). When individuals engage in exercise, skeletal muscle proteins can promote lipid and carbohydrate metabolism (Ryan et al., 2020). Different exercise modalities have been shown to induce a negative energy balance, which reduces fat under the condition that no compensation in other components of energy intake occurs (Thompson et al., 2012). Aerobic exercise (AE; Andreato et al., 2019;Ryan et al., 2020), high-intensity interval training (HIIT; Andreato et al., 2019), and resistance exercise (RE; Keating et al., 2017) are the most commonly used exercise modalities among the general population.

Current guidelines recommend AE as the main training modality to promote metabolic health (Pescatello, 2014) because it can be maintained over an extended period to improve fat mobilization and oxidation (Maillard et al., 2018). During AE, aerobic metabolism activates muscle groups to extract energy in the form of adenosine triphosphate from amino acids, carbohydrates, and fatty acids (Patel et al., 2017). The findings of several meta-analyses indicate that AE reduces substantially the levels of not only subcutaneous adipose tissue (Yarizadeh et al., 2021) but also visceral adipose tissue (VAT; Chang et al., 2021; Sabag et al., 2017). AE interventions have been shown to reduce waist circumference (WC; Armstrong et al., 2022; S. J. Kim et al., 2020) and triglyceride (TG) levels (Baetge et al., 2017) and to increase levels of high-density lipoprotein (HDL; S. J. Kim et al., 2020). HIIT has gained in popularity because it is time efficient, permits various exercise choices, and effectively promotes high levels of excess postexercise oxygen consumption, which increases the body's metabolic rate and helps burn excess calories for hours after exercise (Maillard et al., 2018). Similar to AE, HIIT helps reduce fat mass, body mass index (BMI), WC, and waist-to-hip ratio (WHR; Andreato et al., 2019; Maillard et al., 2018).

RE increases the practitioner's muscular strength and fitness (Yan et al., 2019) but does not significantly reduce fat mass on its own (Bellicha et al., 2021; Chang et al., 2021). Therefore, a combination of AE and RE has been explored as an alternative exercise training strategy (Cronin et al., 2019; Mann et al., 2014). However, the reported effects of this combination have been inconsistent (Alberga et al., 2015; Chang et al., 2021; Yarizadeh et al., 2021). Some studies have claimed that this combination helps reduce more fat mass than does AE alone (Yarizadeh et al., 2021), whereas other studies have reported no superior effects of the combination to those of AE alone in terms of fat mass reduction (Alberga et al., 2015; Chang et al., 2021) or BMI or WC improvement (Lee et al., 2019).

The common metabolic health outcome variables for evaluating the effectiveness of exercise include body composition variables such as VAT (Maillard et al., 2018) and body fat (Ballin et al., 2020); anthropometric parameters such as WC and hip circumference (HC; Baetge et al., 2017; Ballin et al., 2020), WHR (Tsai et al., 2019), body weight (BW; Bellicha et al., 2021), and BMI (Ryan et al., 2020); and lipid profiles such as cholesterol, TG, HDL, and low-density lipoprotein (LDL) levels (H. J. Kim et al., 2020; Ryan et al., 2020; Tsai et al., 2019). These variables are strongly associated with metabolic syndrome (McLaughlin et al., 2019), cardiovascular diseases (Woodward et al., 2020), and all-cause mortality (I. M. Lee et al., 2012). Metabolic health may be promoted by performing adequate exercise, with intensity, duration per session, and frequency as key factors influencing exercise effectiveness (Chang et al., 2021). Current evidence suggests that performing moderate exercise at least 3 times per week for 12 weeks helps improve health outcomes (Chang et al., 2021; Luo et al., 2023; Ryan et al., 2020). However, few studies have comprehensively compared different exercise modalities in terms of effectiveness. Earlier studies have focused primarily on AE–HIIT comparisons (Abdelbasset et al., 2020; Ryan et al., 2020), AE alone, and combinations of AE and RE (Brellenthin et al., 2019; S. Lee et al., 2019).

Although exercise is known to improve both physical and mental health, interventions are difficult to implement (Garner et al., 2018) because of factors including high rates of attrition (Keating et al., 2017) and low levels of adherence (Curtis et al., 2020). In terms of intervention type, both supervised exercise (Christensen et al., 2019) and home-based exercise (Power et al., 2022) are effective. Supervised exercise can facilitate participants' learning efficiency (Ballin et al., 2020), whereas home-based exercise is more accessible and convenient (Ballin et al., 2020) but carries the risk of low adherence (Lund et al., 2019). In earlier studies, the rate of adherence to supervised exercise regimens was found to be 58%–84% (Hwang et al., 2019; A. S. Lee et al., 2020; Picorelli et al., 2014), whereas that for home-based exercise was approximately 50% (Argent et al., 2018). The average attrition rate has been identified as approximately 20%–30% (Keating et al., 2017; S. Lee et al., 2019; Reljic et al., 2018).

Feasibility studies are helpful in evaluating the practicality of implementation and preliminary efficacy before undertaking randomized controlled trials (Vincze et al., 2018), especially multiarmed trials. Although some studies have explored the effects of metabolic health to compare different exercise modalities (Abdelbasset et al., 2020; Zhang et al., 2017), most of this work has been conducted under controlled laboratory conditions (Abdelbasset et al., 2020; Ryan et al., 2020), limiting the generalizability of their findings to the general population. Thus, in this study, a feasibility study is conducted to elucidate the feasibility and preliminary efficacy of different exercise modalities in the clinical field. The findings may serve as a reference for the development of future, formal, randomized controlled trials aimed at identifying the optimal exercise modality for metabolic health promotion among community-dwelling residents.

This feasibility study was conducted to (a) determine the implementation efficacy of supervised and home-based exercise interventions by investigating their respective rates of intervention adherence, adherence to targeted intensity, attrition, and adverse events and (b) explore the preliminary efficacy of 12-week exercise programs among AE, AE combined with RE, and HIIT on body composition, anthropometric parameters, and lipid profiles for community-dwelling residents with physical inactivity.

## Methods

### Design

This randomized controlled trial was composed of four 12-week intervention arms: AE alone, AE combined with RE, HIIT, and the control (nonintervention) group (ClinicalTrials.gov identifier: NCT04496388).

### Participants

This study included physically inactive (engaged in structured exercise for < 3 days/week during the past 3 months) community-dwelling residents aged 40–70 years who were willing to participate. Otherwise, qualified individuals with conditions such as stroke, cardiovascular diseases, pulmonary diseases, disability, and pregnancy that limited their ability to engage in physical exercise were excluded. Individuals with contraindications for use of a body composition analyzer (e.g., wearing a pacemaker, having artificial metal joints or amputated hands or feet, and being unable to be immobilized during the measurement) were also excluded.

### Procedure and Settings

Data collection and intervention implementation were conducted from April to October 2020. Eligible residents were recruited during health examinations conducted at a public health center and through recruitment flyers distributed by village chiefs. Interested individuals were screened for eligibility by the first author, who then explained the research purpose and procedure to eligible individuals and sought their informed consent to participate. Recruitment took place at a public health center and a community center in southern Taiwan.

The participants were randomly assigned to one of the four groups (AE, AE combined with RE, HIIT, and control). Identification numbers for the participants were selected by drawing straws, and block randomization (block size = 4) was performed using the randomly assigned drawing numbers. Group allocation was performed based on the randomization outcomes. Randomization was conducted by a research assistant who was not involved in this study.

The participants in the exercise groups were asked to attend supervised exercise sessions twice a week in the community center and to perform home-based exercises once a week while watching instructional videos. The participants were strangers to one another and had no opportunity to interact with the participants in the other groups. All of the participants followed the intervention assigned to their group only. The participants were instructed to maintain their current dietary intake, and their anthropometric variables, body composition, and blood were examined before and after the 12-week intervention. Although the participants and interveners could not be blinded, the data analyst (first author) was blinded to group allocation.

### Interventions

The contents of the exercise programs were tailored to the participants and designed for moderate to vigorous intensity by two professional physical fitness interveners with > 15 years of experience each in teaching exercise to community residents. All three of the exercise interventions included 10 minutes of warm-up and of cooldown, respectively, before and after the main exercise session (Berge et al., 2021).

The AE group implemented core muscle training composed of side swings, rocking horses, marches, and jumping jacks for 30 minutes continuously. The AE combined with RE group implemented the same 30-minute AE exercise protocol with an additional 10 minutes of RE using free weights or a 25-pound Thera-Band to train the major muscle groups (Su et al., 2022) through leg presses, chest presses, lateral pull-downs, shoulder presses, arm curls, and triceps extensions (Luo et al., 2023). Each session included three to five RE exercises. Exercises for each muscle group were performed in three to five sets of 8–12 repetitions each with a rest interval of 30 seconds between sets to improve muscular fitness.

The HIIT group repeated four sets of training within a 20-minute period, with each set composed of the following eight motions: foot fire, high knee, skaters, scissors, jumping jack, squat jump, front kick, and fast punch. Each motion was performed for 30 seconds and was followed by a 10-second rest. An agility ladder, a 25-pound Thera-Band, a 1-kilogram dumbbell, and a 6-kilogram kettlebell were used to assist participants in training.

To ensure safety and efficacy, the exercises shown in instructional videos for home-based exercise were the same as those shown during supervised exercise. Once each week, the first author followed up with the participants regarding their adherence to home-based exercise when they attended a supervised exercise session. The participants in the control group were instructed to maintain their daily routines and activities, and their activities were monitored once every 2 weeks by the first author using the Line app. All measurements were performed in all four groups before and after the 12-week intervention.

### Measures

#### Body composition analyzer

VAT, BW, and body fat were measured using a body composition analyzer (Accuniq BC300) that uses bioelectrical impedance analysis to estimate body fat and muscle mass distributions. Although no prior studies have reported using the Accuniq BC300 because of its recent launch in 2018, the Accuniq BC360, an identical machine produced by the same manufacturer but with a different weight and appearance, has shown a high reproducibility of 0.997 and correlation coefficients of .95 to dual-energy x-ray absorptiometry (Yang et al., 2018). The exercise group participants fasted for at least 8 hours before the measurements, which were performed the following morning. Before these measurements, the participants were asked to remove their shoes, socks, jewelry, eyeglasses, and any metal objects. BMI was calculated as weight divided by height squared (m^2^).

#### Anthropometric measurements

WC and HC were measured in the standing position using a nonelastic tape and recorded to one decimal place by the first author, who is a well-trained nurse and nurse educator with > 10 years of experience. WC was measured at the midpoint between the lower edge of the rib and the upper edge of the anterior superior iliac spine, whereas HC was measured as the largest circumference around the buttocks (Ma et al., 2022; Tsai et al., 2019). The WHR was derived by dividing WC by HC (Tsai et al., 2019).

#### Blood samples

Blood samples for lipid profiles, including cholesterol, TG, HDL, and LDL levels, were collected after an overnight fast of ≥ 8 hours. After collection, the samples were stored at 2°C–15°C and then centrifuged for 7 minutes at 3,000 rpm for analyses.

#### Heart rate monitor watch

A Xiaomi Mi Band 3 was used to monitor exercise intensity during each session by measuring the participants' heart rate within 1 minute of completing the exercise. The targeted exercise intensity for participants in the AE, AE combined with RE, and HIIT groups was ≥ 40% of the heart rate reserve (HRR) to ensure moderate to vigorous intensity was reached (Pescatello, 2014). In this study, the correlation coefficient between the Xiaomi Mi Band 3 and the 60-second radial pulse, as checked by the first author, was .80.

#### Demographic and clinical characteristics of the participants

Data regarding the demographic and clinical characteristics of the participants were collected using a questionnaire developed by the first author. The collected data included information on gender, age, marital status, educational level, employment status, smoking status, alcohol consumption, and chronic diseases.

### Ethical Considerations

Ethical approval was obtained from the human research ethics committee of a university in southern Taiwan (No. 109-035).

### Data Analysis

Data were analyzed using SPSS Version 24.0 (IBM Inc., Armonk, NY, USA). Descriptive statistics such as frequency, percentage, mean, and standard deviation were used to analyze the demographic and clinical characteristics of the participants. An analysis of variance and a chi-square test were performed to make between-group comparisons of baseline demographic and clinical characteristics. The likelihood ratio chi-square test was used when a data set was too small to meet the requirement that > 80% of cells in the cross table have expected values of ≥ 5 (Abbas et al., 2022). The within-group differences were compared using a paired *t* test. A general linear model with Helmert contrasts was used to examine significant between-group differences in mean values after controlling for baseline values (Chien et al., 2015). Effect sizes were measured using eta squared (η^2^), which represents the proportion of variance in the dependent variable explained by a predictor (J. Wang et al., 2020). η^2^ values of .01, .06, and .14 were defined as small, medium, and large effects, respectively (J. Wang et al., 2020).

## Results

**Figure 1.**
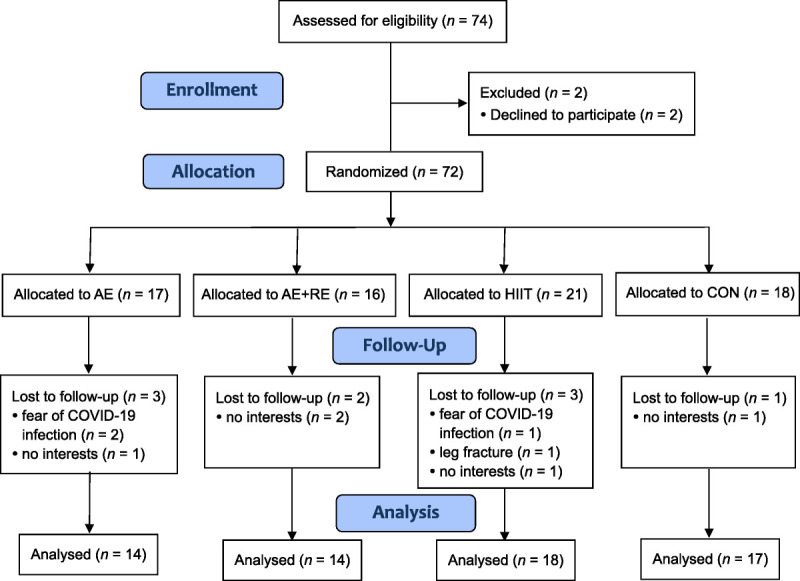
Flowchart of Study Participation *Note.* AE = aerobic exercise; RE = resistance exercise; HIIT = high-intensity interval training; CON = control group.

Of the 74 residents approached, 72 were enrolled and randomized into the AE (*n* = 17), AE combined with RE (*n* = 16), HIIT (*n* = 21), and control (*n* = 18) groups (see Figure 1). Most of the participants were women (88.9%), retired (65.3%), and married (69.4%). The mean age was 58.6 years. No significant intergroup differences were noted in terms of demographic or clinical characteristics (Table 1).

**Table 1.** Demographic and Clinical Characteristics of the Participants Allocated Among the Four Study Arms (*N* = 72)

Variable	AE (*n* = 17)		AE + RE (*n* = 16)		HIIT (*n* = 21)		CON (*n* = 18)		χ^2^	*p*
	*n*	%	*n*	%	*n*	%	*n*	%		
Gender									2.39 ^a^	.495
Male	1	5.9	2	12.5	4	19.0	1	5.6		
Female	16	94.1	14	87.5	17	81.0	17	94.4		
Age (years; *M* and *SD*)	59.59	7.03	58.19	8.16	57.71	8.72	59.06	7.48	0.21 ^b^	.889
< 65	11	64.7	11	68.8	16	76.2	13	72.2		
≥ 65	6	35.3	5	31.3	5	23.8	5	27.8		
Marital status									6.86 ^a^	.077
Unmarried	4	23.5	3	18.7	5	23.8	10	55.6		
Married	13	76.5	13	81.3	16	76.2	8	44.4		
Educational level									1.07 ^a^	.983
≤ Junior high school	3	17.7	2	12.5	3	14.3	4	22.2		
Senior high school	5	29.4	5	31.3	7	33.3	4	22.2		
≥ College	9	52.9	9	56.2	11	52.4	10	55.6		
Employment status									11.42 ^a^	.076
Unemployment	10	58.8	12	75.0	14	66.6	11	61.1		
Part-time	2	11.8	3	18.7	1	4.8	6	33.3		
Full-time	5	29.4	1	6.3	6	28.6	1	5.6		
Smoking									4.01 ^a^	.261
No	17	100.0	16	100.0	19	90.5	17	94.4		
Yes	0	0.0	0	0.0	2	9.5	1	5.6		
Alcohol consumption									2.36 ^a^	.502
No	16	94.1	16	100.0	19	90.5	17	94.4		
Yes	1	5.9	0	0.0	2	9.5	1	5.6		
Chronic disease										
Hypertension	2	11.8	4	25.0	4	19.0	4	22.2	1.11 ^a^	.776
Diabetes mellitus	2	11.8	1	6.3	2	9.5	3	16.7	1.01 ^a^	.800
Cancer	2	11.8	2	12.5	1	4.8	2	16.0	0.96 ^a^	.812
Metabolic syndrome	3	17.6	5	31.3	7	33.3	4	22.2	1.58 ^a^	.664

*Note.* AE = aerobic exercise; RE = resistance exercise; HIIT = high-intensity interval training; CON = control group.

^a^ Likelihood ratio chi-square test. ^b^ Analysis of variance test.

The total adherence rates to exercise frequency, including supervised and home-based exercise, were 70.84%, 86.29%, and 75.71% in the AE, AE combined with RE, and HIIT groups, respectively. Rates of adherence to supervised exercise (74.01%, 87.54%, and 80.44%, respectively; *p* = .409) and home-based exercise (64.98%, 83.90%, and 65.50%, respectively; *p* = .135) did not significantly differ among the groups (Table 2). In each supervised exercise session, rates of adherence to reaching the targeted level of exercise intensity, as indicated by the HRR, were 92.65%, 90.50%, and 82.65% in the AE, AE combined with RE, and HIIT groups, respectively (*p* = .343; Table 2).

**Table 2.** Adherence to Supervised Exercise, Home-Based Exercise, and Targeted Exercise Intensity and Attrition Rate Among the Three Exercise Groups

Variable	AE (*n* = 14)	AE + RE (*n* = 14)	HIIT (*n* = 18)	*F*	*p*
	%	%	%		
Adherence to supervised and home-based exercise	70.84	86.29	75.71	1.55	.224
Adherence to supervised exercise	74.01	87.54	80.44	0.91	.409
Adherence to home-based exercise	64.98	83.90	65.50	2.09	.135
Adherence to targeted intensity of exercise	92.65	90.50	82.65	1.09	.343
Attrition rate	17.65	12.50	14.29	0.41	.749

*Note.* AE = aerobic exercise; RE = resistance exercise; HIIT = high-intensity interval training.

The total attrition rate was 12.5% (*n* = 9), with no significant difference (*p* = .749) found among the groups (17.65%, 12.50%, 14.29%, and 5.56% in the AE, AE combined with RE, HIIT, and control groups, respectively; Figure 1 and Table 2). No adverse events related to the exercise intervention were reported. One participant in the HIIT group sustained a leg fracture during the study period while descending a staircase, but this event was unrelated to the exercise intervention.

The mean changes in body composition, anthropometric parameters, and lipid profiles between baseline and posttest are presented in Table 3. In the HIIT group, BW (−0.56 [1.11], *p* = .048), BMI (−0.22 [0.40], *p* = .03), WC (−2.61 [3.54], *p* = .006), cholesterol (−24.67 [38.04], *p* = .014), and LDL (−13.61 [25.56], *p* = .037) all significantly decreased. However, VAT (8.83 [11.32], *p* = .004), body fat (1.86 [2.61], *p* = .008), and WHR (0.02 [0.03], *p* = .025) significantly increased.

**Table 3.** Changes in Outcomes After 12 Weeks of Each Exercise Modality

Variable	AE (*n* = 14)		AE + RE (*n* = 14)		HIIT (*n* = 18)		CON (*n* = 17)	
	△Change	*p*	△Change	*p*	△Change	*p*	△Change	*p*
Body composition								
VAT (cm^2^)	7.21 (26.03)	.319	5.50 (11.26)	.091	8.83 (11.32)	.004	8.82 (32.06)	< .001
Body fat (%)	1.46 (5.23)	.314	2.11 (4.31)	.090	1.86 (2.61)	.008	2.04 (2.41)	.003
Anthropometric parameters								
BW (kg)	0.05 (1.12)	.870	−0.50 (0.94)	.069	−0.56 (1.11)	.048	0.33 (0.96)	.178
BMI (kg/m^2^)	0.06 (0.57)	.714	−0.19 (0.39)	.100	−0.22 (0.40)	.030	0.12 (0.37)	.186
WC (cm)	−2.68 (3.33)	.010	−2.18 (5.82)	.185	−2.61 (3.54)	.006	−1.50 (8.82)	.493
HC (cm)	−1.11 (2.74)	.154	0.25 (4.25)	.829	−0.71 (3.35)	.384	1.12 (2.77)	.116
WHR	0.01 (0.05)	.296	0.01 (0.03)	.060	0.02 (0.03)	.025	0.02 (0.03)	.002
Lipid profiles								
Cholesterol (mg/dl)	−8.50 (32.06)	.339	−3.29 (14.77)	.420	−24.67 (38.04)	.014	−1.65 (37.09)	.857
TG (mg/dl)	6.71 (25.49)	.342	−9.29 (31.68)	.293	−18.78 (119.15)	.513	−24.29 (121.55)	.422
HDL (mg/dl)	−0.21 (6.09)	.897	1.71 (9.60)	.516	−1.00 (11.45)	.716	−0.76 (8.52)	.716
LDL (mg/dl)	−8.21 (25.66)	.252	−10.11 (26.52)	.177	−13.61 (25.56)	.037	6.71 (32.57)	.409

*Note.* △Change = Mean of Postexercise Training − Mean of Baseline Response. A paired *t* test was used to compare within-group differences. AE = aerobic exercise; RE = resistance exercise; HIIT = high-intensity interval training; CON = control group; VAT = visceral adipose tissue; BW = body weight; BMI = body mass index; WC = waist circumference; HC = hip circumference; WHR = waist-to-hip ratio; TG = triglycerides; HDL = high-density lipoprotein; LDL = low-density lipoprotein.

The between-group differences in terms of Helmert contrasts are presented in Table 4. The postintervention BW (*p* = .048) and LDL levels (*p* = .02) in all three exercise groups were significantly lower than those in the control group, but the efficacy of BW and LDL in HIIT was not superior to the AE alone or AE combined with RE groups. The η^2^ values for BW and LDL were .109 and .097, indicating a moderate effect size.

**Table 4.** Between-Group Differences in Change in Outcomes After 12 Weeks Using the Helmert Contrast Test

Variable/Group Comparison	Helmert Contrasts		
	Contrast Estimation	*p*	η^2^
Body composition			
Visceral adipose tissue (cm^2^)			.005
CON vs. HIIT, AE + RE, AE	0.362	.933	
HIIT vs. AE + RE, AE	2.223	.621	
AE + RE vs. AE	−1.189	.833	
Body fat (%)			.004
CON vs. HIIT, AE + RE, AE	0.007	.994	
HIIT vs. AE + RE, AE	−0.295	.739	
AE + RE vs. AE	−0.378	.735	
Anthropometric parameters			
Body weight (kg)			.109
CON vs. HIIT, AE + RE, AE	0.602	.048	
HIIT vs. AE + RE, AE	−0.298	.345	
AE + RE vs. AE	−0.544	.169	
Body mass index (kg/m^2^)			.111
CON vs. HIIT, AE + RE, AE	0.211	.092	
HIIT vs. AE + RE, AE	−0.161	.217	
AE + RE vs. AE	−0.252	.125	
Waist circumference (cm)			.004
CON vs. HIIT, AE + RE, AE	0.476	.743	
HIIT vs. AE + RE, AE	0.111	.943	
AE + RE vs. AE	0.714	.710	
Hip circumference (cm)			.045
CON vs. HIIT, AE + RE, AE	1.097	.243	
HIIT vs. AE + RE, AE	−0.157	.871	
AE + RE vs. AE	1.378	.256	
Waist-to-hip ratio			.007
CON vs. HIIT, AE + RE, AE	0.002	.803	
HIIT vs. AE + RE, AE	0.006	.536	
AE + RE vs. AE	−0.001	.941	
Lipid profiles			
Cholesterol (mg/dl)			.069
CON vs. HIIT, AE + RE, AE	12.250	.122	
HIIT vs. AE + RE, AE	−10.336	.226	
AE + RE vs. AE	1.567	.880	
Triglycerides (mg/dl)			.038
CON vs. HIIT, AE + RE, AE	−2.910	.859	
HIIT vs. AE + RE, AE	21.667	.226	
AE + RE vs. AE	−18.236	.402	
High-density lipoprotein (mg/dl)			.023
CON vs. HIIT, AE + RE, AE	−0.592	.817	
HIIT vs. AE + RE, AE	−2.423	.376	
AE + RE vs. AE	2.558	.453	
Low-density lipoprotein (mg/dl)			.097
CON vs. HIIT, AE + RE, AE	16.539	.020	
HIIT vs. AE + RE, AE	−0.180	.981	
AE + RE vs. AE	−5.741	.534	

*Note.* CON = control group; HIIT = high-intensity interval training; AE = aerobic exercise; RE = resistance exercise.

## Discussion

In this feasibility study, exercise interventions were implemented in a population of community-dwelling adults at adherence rates of 74.01%–87.54% and 64.98%–83.90% for completing supervised and home-based exercise, respectively, with an 82.65%–92.65% adherence to target exercise intensity, low attrition rate (12.5%), and no adverse events. The preliminary findings related to efficacy indicate HIIT reduces BW, BMI, WC, cholesterol, and LDL after 12 weeks. Furthermore, BW and LDL decreased significantly in all three exercise groups after 12 weeks compared with the control group, although the intergroup differences were not significant among exercise groups.

Evidence reported in the literature indicates that exercising at least three 3 a week (Chang et al., 2021; Pescatello, 2014) with ≥ 80% adherence is necessary to improve health outcomes (Arem et al., 2016; Lund et al., 2019). However, in this study, the 12-week exercise intervention showed positive outcomes in BW and LDL only, indicating that the relatively low rate of adherence to home-based exercise (64.98%–83.90%) was insufficient to improve other metabolic health outcomes (e.g., VAT, body fat, WC, HC, cholesterol, TG, and HDL). Although the rate of adherence to overall exercise frequency in this study was higher than that reported in prior studies (58%–84% for supervised exercise and 50% for home-based exercise; Argent et al., 2018; Hwang et al., 2019; A. S. Lee et al., 2020; Picorelli et al., 2014), room for improvement remains. The strong support given to this 12-week intervention by related stakeholders (e.g., village and neighborhood chiefs) and the high ratio of retirees (65.3%) in the study sample may significantly explain the high adherence rate in this study. Before the intervention, village chiefs encouraged residents to participate and answered their potential concerns, improving the exercise adherence rate. We recommend that future studies cooperate with stakeholders in communities and include at least three supervised exercise sessions per week in addition to one home-based exercise session.

The satisfactory adherence to target exercise intensity and the lack of adverse events related to exercise achieved in this study indicate these interventions as suitable for application with the study population. Although no significant differences were noted after age-based stratification (< 65 years and ≥ 65 years) in terms of adherence to target exercise intensity (*t* = 0.31, *p* = .758) or changes in body composition, anthropometric parameters, or lipid profiles in this study, we noted that, in the beginning, the interveners found designing the exercise intervention challenging in terms of intensity because of the significant heterogeneity in participant ages (40–70 years). Given that the basal metabolic rate declines at 1%–2% per decade (Roberts & Rosenberg, 2006), age distribution should be expected to influence the effect of exercise on study outcomes. In the future, studies should design exercise interventions separately for adults younger and older than 65 years that take into consideration their physiological capabilities (Pescatello, 2014). Moreover, in addition to measuring exercise intensity objectively using HRR, subjective physical exertion may also be considered to facilitate the further tailoring of exercise intensity to personal physical fitness level.

The findings of this study were inconsistent with a previous study that found HIIT to lead to a higher reduction in LDL levels than AE (Vella et al., 2017). Reductions in LDL levels are strongly affected by the level of decrease in BW (Brown et al., 2016; Siri-Tarino et al., 2009). Brown et al. reported that participants with obesity who lost > 10% of their BW exhibited higher reductions in LDL than their peers who lost 5%–10% and < 5% of their BW. In this study, although the HIIT group exhibited higher reductions in BW and BMI than either the AE alone or AE combined with RE groups, the differences were not significant. No participants in the HIIT group lost > 10% of their BW. The relatively low reductions in BW reported in this study may explain the nonsignificant differences between the HIIT and other groups in terms of changes in lipid profiles (Y. Wang & Xu, 2017). The factors that influence lipid profiles may more significantly impact the relationship between dose–response and energy expenditure than exercise modality (Mann et al., 2014). These results should be further explored and confirmed in future studies using larger sample sizes.

This study found that VAT and body fat, where excessive energy is preferentially stored and directly influenced by the hepatic and intestinal metabolism (Chang et al., 2021), increased after 12 weeks in all three exercise groups, implying that the qualitative aspects of diet and dietary patterns during the intervention period may greatly influence the results. Although the participants were not asked to record their diet, we found they tended to eat more dessert-type foods after exercise sessions because they felt hungry or required compensation for their energy consumption. Some participants claimed that the COVID-19 outbreak had led them to spend more time at home rather than on outdoor physical activities. They also claimed that their dietary intake was higher than usual. This phenomenon was observed in all three exercise groups, which may explain why both their VAT and body fat increased after exercise (H. J. Kim et al., 2020). Thus, we strongly suggest that participants be provided nutritional education before and after each exercise session. In particular, HIIT leads to increases in the postexercise metabolic rate and associated fat expenditure during the recovery period (Zhang et al., 2017). Consuming appropriate nutritional supplements rather than increasing caloric intake after exercise sessions may further improve the efficacy of the HIIT intervention.

Except for the warm-up and cool-down, the durations of each AE (30 minutes) and HIIT (20 minutes) session were consistent with most studies (Chang et al., 2021; Chin et al., 2020; Ryan et al., 2020). In the AE combined with RE group, the duration of the additional RE varied between previous studies (Bellicha et al., 2021; Chang et al., 2021). Studies have compared AE alone and AE combined with RE under two prescription designs: same AE protocol with additional RE or a shorter AE protocol with a longer duration of additional RE (Chang et al., 2021; Cuff et al., 2003). Both approaches are feasible when energy consumption is sufficient (Chang et al., 2021). We used the same AE protocol with additional RE intervention design because we intended to explore whether additional RE combined with AE was more effective than AE alone. However, using precise physiological equipment to ensure similar energy consumption and explore the metabolic health effects between exercise modalities in the community residents was difficult. Instead, for practical purposes, we attempted to ensure that the three exercise groups had similar adherence rates in terms of reaching at least moderate-intensity exercise per session. The results revealed that these interventions efficiently reduced BW and LDL in all of the exercise groups, indicating the feasibility of the proposed exercise intervention programs.

### Implications

The findings of this study have implications for nursing practice as well as future research. Regarding the implications for nursing practice, the successful implementation of this study illustrates the feasibility of applying multiple arms with different exercise modalities on groups of community-dwelling residents. We observed that a 12-week exercise intervention is sufficient to efficiently reduce BW and LDL regardless of type of exercise. Thus, nurses may encourage community residents to perform at least moderate exercise 3 times a week for 12 weeks.

In terms of implications for future research, several refinements of this study are proposed to overcome its limitations. First, dietary education should be provided before and after each exercise session to prevent/reduce participants' postsession consumption of compensatory high-calorie foods that may reduce or cancel the positive effects of exercise. Second, the exercise effects may be stratified by age group (e.g., younger and older than 65 years), and the supervised sessions may be implemented 3 times per week in addition to one home-based session. A more homogenous age group can facilitate the design of courses better suited to the participants' physiological capabilities. For participants without significant improvements in metabolic health outcomes at the current dosage, multiple adaptive exercise interventions may be attempted in the future.

### Limitations

This study was affected by several limitations. First, as this was designed as a feasibility study, we did not include BMI or body fat percentage limitations in the inclusion criteria. Further studies may restrict the inclusion criteria to individuals at a high risk of cardiovascular diseases (e.g., being overweight or obese) to explore their postexercise health outcomes. Second, the preliminary efficacy results of this study should be interpreted/generalized with caution because of the small sample sizes in each arm. However, the η^2^ indicates moderate effects on BW and LDL. Furthermore, the high percentage of female participants in this study may influence the findings (Dalah et al., 2021). The voluntary nature of participation indicates women may be more eager to participate in community activities than men in Taiwan. Future studies should explore strategies for improving the rate of male participation in community activities. Third, both supervised and home-based exercise regimens were included in our study; future studies may focus on comparing between the regimens to identify the optimal strategy. Finally, the lack of precise physiological monitoring equipment to measure maximum oxygen consumption (VO_2max_) made it difficult to precisely compare energy consumption among the three exercise groups. However, no significant difference among these groups was found using HRR to monitor whether participants reached the target exercise intensity.

### Conclusions

The results of this study support the feasibility and efficacy of implementing well-designed exercise interventions on community-dwelling residents in southern Taiwan. The participants exhibited acceptable-to-good adherence to supervised exercise and home-based exercise programs, satisfactory adherence to the targeted level of exercise intensity, low rates of attrition, and no adverse events. HIIT was found to significantly reduce BW, BMI, WC, cholesterol, and LDL after 12 weeks. A 12-week, thrice-weekly intervention of any of the three exercise modalities can efficiently decrease BW and LDL in community-dwelling residents as long as ≥ 82.65% rate of adherence to the moderate-intensity exercise and > 70.84% rate of adherence to supervised and home-based exercise are maintained.
